# Patient-specific instrumentation for total knee arthroplasty improves reproducibility in the planned rotational positioning of the tibial component

**DOI:** 10.1186/s13018-022-03298-9

**Published:** 2022-09-05

**Authors:** Masaichi Sotozawa, Ken Kumagai, Shunsuke Yamada, Shuntaro Nejima, Yutaka Inaba

**Affiliations:** Department of Orthopaedic Surgery, Graduate School of Medicine, Yokohama City University Hospital, Yokohama City University, 3-9 Fukuura, Kanazawa-ku, Yokohama, 236-0004 Japan

**Keywords:** Total knee arthroplasty, Patient-specific instrumentation, Conventional method, Tibial rotational alignment

## Abstract

**Background:**

The purpose of this study was to evaluate the reproducibility of planned component positioning including tibial rotational alignment in patient-specific instrumentation (PSI) for total knee arthroplasty (TKA).

**Methods:**

A total of 100 knees of 100 patients underwent TKA using PSI (*n* = 50) or the conventional method (*n* = 50). Full-length anteroposterior radiographs of the lower limb were taken in the standing position, and the coronal alignments of the femoral and tibial components were measured. Computed tomography (CT) images of the lower limb were obtained preoperatively and postoperatively, and the rotational alignments of the femoral and tibial components were measured. The difference from the preoperative planning in tibial rotational alignment was measured using three-dimensionally merged pre- and postoperative images. The mean values and rates of outliers in each measurement were compared between the PSI group and the conventional group.

**Results:**

There were no significant differences in coronal alignment of the femoral and tibial components and rotational alignment of the femoral component between the two groups. With respect to rotational alignment of the tibial component from the preoperatively planned reference axis, the PSI group showed a lower rate of outliers (internal rotation > 10°) than the conventional group (*p* < 0.05).

**Conclusions:**

This study demonstrated that the difference from the preoperative planning in tibial rotational positioning was accurately evaluated using novel three-dimensional measurement method, and PSI could reduce outliers in rotational alignment of the tibial component (internal rotation > 10°). PSI is a useful technique for improving the reproducibility of the planned tibial rotational positioning in TKA.

## Introduction

Total knee arthroplasty (TKA) is an established surgical procedure to treat end-stage knee osteoarthritis. Accurate bone cutting and restoration of mechanical limb alignment are the important factors affecting long-term survival. Coronal malalignment is the common cause of revision surgery [1–3]. Rotational alignment of the components is also important for successful TKA. Malrotation of either the femoral or tibial component can lead to patellar subluxation or dislocation [4] and unexplained knee pain [5, 6]. Unexpected malalignment and malposition of the components are a problem for orthopedic surgeons performing TKA.

The conventional 2D planning-based technique using intramedullary or extramedullary alignment guides is still popular for bone cutting and component positioning in TKA. A meta-analysis reported that mechanical axis malalignment of greater than 3° occurred in a mean of 31.8% of patients who underwent conventional TKA [7]. Optimal placement of the prosthetic components is a major issue in TKA, and computer-assisted surgery or patient-specific instrumentation (PSI) has been developed to improve limb and prosthesis alignment and optimal positioning of the components.

PSI for TKA is a technique that provides 3D templating and custom-fit cutting guides of the distal femur and proximal tibia for each patient using bone models based on computed tomography (CT) or magnetic resonance imaging. This system helps reduce operative time and the number of instrumentation trays [8, 9]. PSI is expected to increase the accuracy of component positioning and reduce outliers [8, 10, 11]. Many studies have investigated the coronal and sagittal alignment of the components using PSI. However, there are few reports evaluating the effect of PSI on rotational alignment in the transverse plane, especially in tibial rotation [12]. In addition, these previous studies evaluated the rotational alignment of the tibial component using only postoperative CT images [13, 14], and it was unknown whether the component was positioned as preoperatively planned. Thus, the present study investigated the accuracy of the rotational positioning in the tibial component using the same reference axis as the preoperative planning.

The purpose of this study was to evaluate the reproducibility of component positioning including tibial rotational alignment in PSI technique for TKA using the same reference axis as the preoperative planning. It was hypothesized that PSI improves the reproducibility of the planned component positioning and reduces outliers compared to the conventional technique.

## Materials and methods

### Patients

This retrospective, case–control study was approved by the institutional review board (#B200500006), and written, informed consent was obtained from all patients. Inclusion criteria for this study were patients with osteoarthritis or rheumatoid arthritis of the knee who underwent primary cemented cruciate-retaining TKA (Vanguard™ Complete Knee System; Zimmer Biomet Inc., Warsaw, IN, USA) without resurfacing the patella by several surgeons between 2018 and 2020. Exclusion criteria were patients with a history of osteotomy, valgus knee deformity, or post-traumatic osteoarthritis. A total of 100 knees of 100 patients were enrolled in this study. PSI was used in 50 consecutive knees, and the conventional method was used in 50 consecutive knees.

### Preoperative planning and surgical procedure

Preoperative planning and the surgical procedure were performed according to the previously described methods [15, 16]. For the PSI group, CT of the lower limb through the hip and ankle was performed preoperatively using the Signature™ system manufacturing protocol. Virtual 3D models of the femur and tibia were created based on CT data with a specific software program, and the patient-specific cutting jigs were generated based on the surgeon’s predetermined default settings: femur varus/valgus 0° (perpendicular to the mechanical axis), flexion 3°, rotation 0° (parallel to the transepicondylar axis); tibia varus/valgus 0° (perpendicular to the mechanical axis), posterior slope 3°, and rotation 0° (line connecting the medial third of the tibial tuberosity and the center point of the attachment of the posterior cruciate ligament).

A standard anterior midline skin incision was made, and the knee joint was exposed through a medial parapatellar arthrotomy. A measured resection technique was used in all patients. For the PSI group, patient-specific cutting guides were carefully placed over the articular surfaces prior to removing osteophytes, and positioning guide pins were inserted through the pin holes. For the conventional group, traditional jig-based instrumentation was used. Target alignment of component positioning was similar to the setting in the PSI group. The tibial anterior–posterior (AP) axis (rotation 0°) was visually determined with reference to the line connecting the medial third of the tibial tuberosity and the center of the attachment of the posterior cruciate ligament, and as a positioning guide, a Kirschner wire was inserted parallel to this reference line just below the bone cutting level prior to bone resection. The bone cuts were made through the slot on the standard resection blocks using traditional saw blades. Femoral and tibial components were inserted with bone cement.

### Measurements of coronal alignment

Full-length anteroposterior radiographs of the lower limb were taken in the standing position, and the *α* angle and *β* angle were measured according to the method reported by Ewald [17]. The *α* angle was defined as the angle between the femoral functional axis and the articular surface of the femoral component. The *β* angle was defined as the angle between the functional axis of the tibia and the articular surface of the tibial component. Outliers were defined as deviations of more than 3° either varus or valgus from the targeted neutral component positioning [15, 18].

### Measurements of rotational alignment using CT-based simulation software

**Fig. 1:**
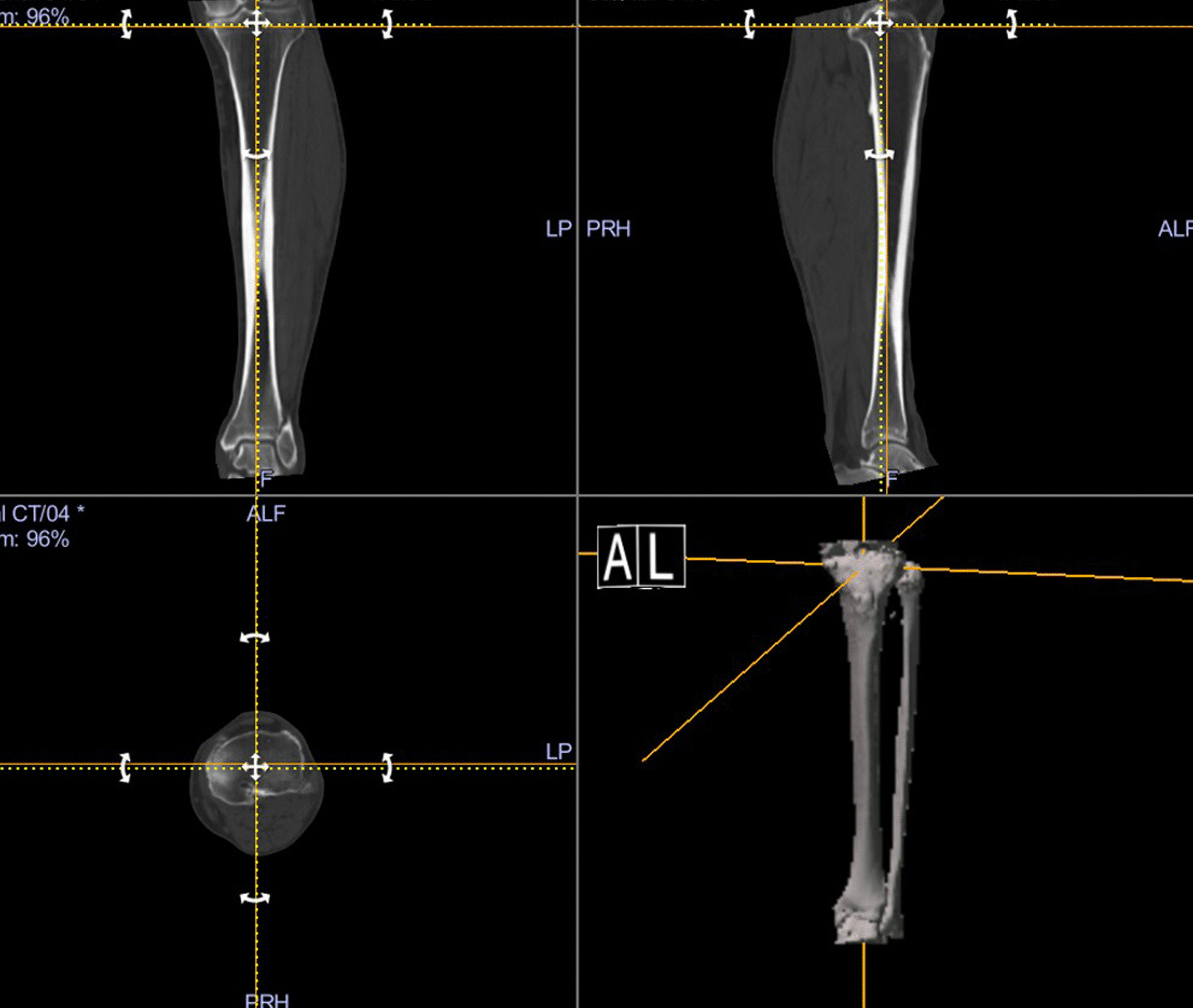
Coronal and sagittal axes are determined using the line passing through the centers of the knee and ankle joints

**Fig. 2:**
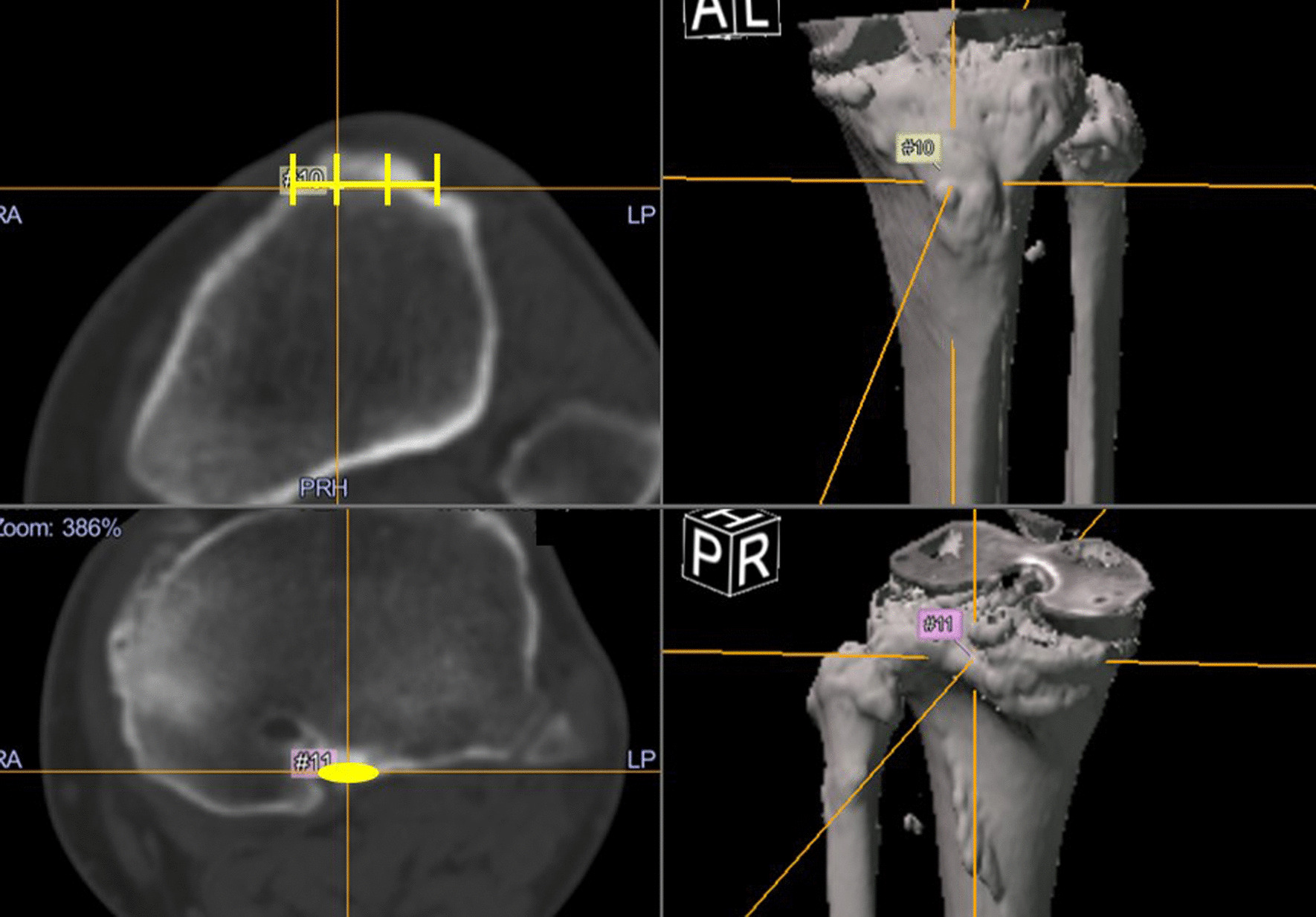
The anteroposterior axis is defined as the line connecting the two points of the tibia. The point is the medial third of the tibial tuberosity, and the other point is the center of the posterior cruciate ligamentous insertion

**Fig. 3:**
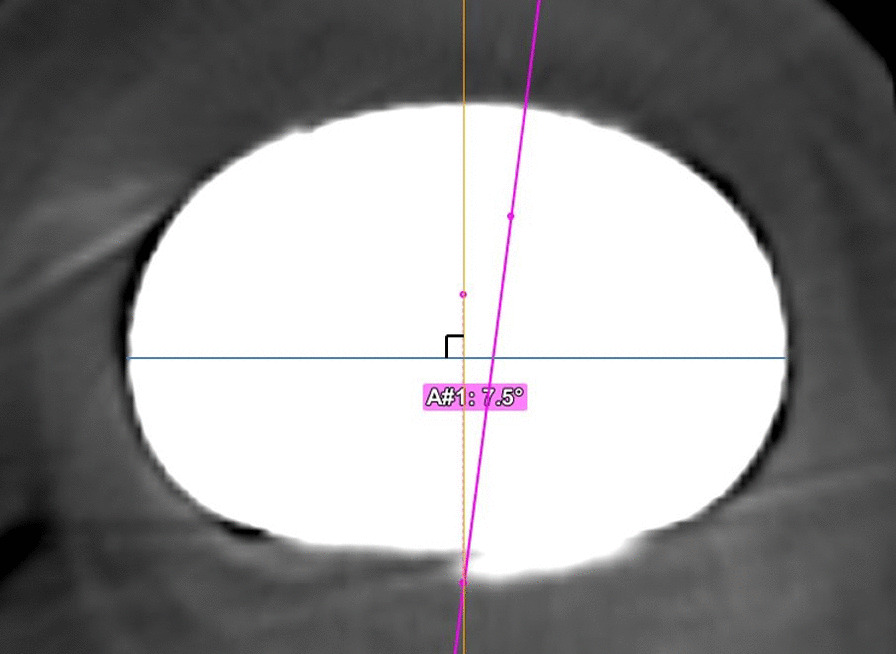
The tibial component axis is defined as the line perpendicular to the major axis of the implant. The tibial rotation angle is the angle between the tibial component axis and the tibial anteroposterior axis

CT images of the lower limb were obtained preoperatively and postoperatively, and measurements of rotational alignment were performed using Orthomap 3D software (Stryker Corporation, Kalamazoo, MI, USA). The measurement of femoral rotational alignment was carried out using the protocol previously described by Berger et al. [4]. A single axial plane of the femur was used at the level of the femoral epicondyles. The surgical transepicondylar axis was drawn from the sulcus of the medial epicondyle to the prominence of the lateral epicondyle. The posterior condylar line of the femoral component was drawn connecting the posterior margins of the medial and lateral posterior component condylar surfaces. The angle between these two lines was measured as the femoral rotational alignment. Outliers of femoral rotational alignment were defined as deviation of more than 3° either internally or externally from the targeted neutral component positioning [18, 19]. For measurement of the difference from the preoperative planning in tibial rotational alignment, the preoperative CT images were superimposed on the postoperative CT images. On the preoperative CT image, the line connecting the center of the knee joint and the center of the ankle joint was set as the *z*-axis (Fig. 1). The reference axis of tibial rotation was set as the line connecting the medial third of the tibial tuberosity and the center point of the attachment of the posterior cruciate ligament, and this line was also used as the reference axis of rotation during the TKA procedure (Fig. 2). The base of the tibial component was set as the *xy* plane on the postoperative CT image. The anteroposterior (AP) axis of the component was defined as the line perpendicular to the major axis of the implant. By superimposing the preoperative and postoperative CT images, the angle between the reference axis of tibial rotation and the AP axis of the tibial component was measured as the tibial rotational angle (Fig. 3). Outliers of tibial rotational alignment were defined as deviation of internal rotation of greater than 10° from the targeted neutral component positioning [6].

### Statistical analysis

Statistical analysis was carried out using IBM SPSS Statistics (version 27.0; IBM Corporation, Chicago, IL, USA). Normality of the data distributions was assessed using the Shapiro–Wilk test. Student’s *t *test or the Mann–Whitney U test was used to compare continuous variables between two groups. Pearson’s chi-squared test was used to compare categorical variables. A *p* value < 0.05 was considered significant. The intra-rater and inter-rater reliabilities of measurements were assessed by calculating intraclass correlation coefficients (ICCs).

## Results

### Patients’ demographics

There were no significant differences between the PSI group and the conventional group in age, sex, body mass index, and hip–knee–ankle angle (Table 1).

**Table 1:** Patients’ demographic data

	PSI group (*n* = 50)	Conventional group (*n* = 50)	*p* value
Gender, male/female	12/38	11/39	0.812
Mean age, years	74.7 ± 9.0	74.7 ± 8.1	0.948
Height, cm	154.8 ± 8.9	154.0 ± 8.5	0.912
Weight, kg	64.5 ± 13.3	63.4 ± 13.2	0.924
Body mass index, kg/m^2^	26.5 ± 4.6	26.4 ± 4.3	0.545
Preoperative hip–knee–ankle angle, °	− 10.3 ± 6.1	− 10.4 ± 7.1	0.658

Values are number or mean ± standard deviation

### Radiographic assessment of coronal alignment

The postoperative values of the *α* angle and the *β* angle were 88.8 ± 2.0° and 90.1 ± 2.4° in the PSI group and 89.2 ± 2.1° and 88.9 ± 1.8° in the conventional group, respectively (Table 2). The number of outliers (> ± 3°) for the *α* angle and *β* angle was 9 (18%) and 6 (12%) in the PSI group and 6 (12%) and 10 (20%) in the conventional group, respectively (Table 2). No significant differences were found in these values.

**Table 2:** Coronal alignment of femoral and tibial components

	PSI group (*n* = 50)	Conventional group (*n* = 50)	*p* value
Mean value			
*α* angle	88.8 ± 2.0	89.2 ± 2.1	0.16
*β* angle	90.1 ± 2.4	88.9 ± 1.9	0.83
Outliers out of ± 3°			
*α* angle	9 (18%)	6 (12%)	0.40
*β* angle	6 (12%)	10 (20%)	0.28

Values are number (percentage) or mean ± standard deviation

### CT-based assessment of rotational alignment

The mean value of femoral rotation was − 1.5 ± 2.5° in the PSI group and − 0.4 ± 3.4° in the conventional group (n.s, Table 3). The number of outliers (> ± 3°) for femoral rotation was 13 (26%) in the PSI group and 15 (30%) in the conventional group (n.s, Table 3). The mean value of tibial rotation from the preoperatively planned reference axis was − 2.6 ± 6.3° in the PSI group and − 4.7 ± 8.1° in the conventional group (n.s, Table 3). The number of outliers (< − 10°) for tibial rotation was 6 (12%) in the PSI group and 15 (30%) in the conventional group (*p* < 0.05, Table 3).

**Table 3:** Rotational alignment of femoral and tibial components

	PSI group (*n* = 50)	Conventional group (*n* = 50)	*p* value
Femoral rotation			
Mean value	− 1.5 ± 2.5	− 0.4 ± 3.4	0.11
Outliers out of ± 3°	13 (26%)	15 (30%)	0.66
Tibial rotation			
Mean value	− 2.6 ± 6.3	− 4.7 ± 8.1	0.16
Outliers < − 10°	6 (12%)	15 (30%)	0.027

Values are number (percentage) or mean ± standard deviation

### Reproducibility of measurements

The ICCs for inter-rater reliabilities by two independent observers were 0.88 for the *α* angle, 0.95 for the *β* angle, 0.83 for femoral rotation, and 0.94 for tibial rotation. The ICCs for intra-rater reliabilities at more than 2-month intervals were 0.84 for the *α* angle, 0.92 for the *β* angle, 0.82 for femoral rotation, and 0.93 for tibial rotation.

## Discussion

The most important finding of the present study was that PSI enabled the reduction of rotational malalignment of the tibial component, which was defined as internal rotation > 10°. However, no significant differences were found in the other parameters between PSI and the conventional technique. These findings partially confirm the initial hypothesis that PSI improves component positioning and reduces outliers compared to the conventional technique. The major advance in this study was the accurate measurement of the difference from the preoperative planning in tibial rotational alignment using three-dimensionally merged images with preoperative ones.

This study focused on evaluation of tibial rotational positioning, since it has been less reported than rotational alignment of the femoral component. Several studies evaluated tibial rotational alignment in the transverse plane of the bone resection level [4, 20–22], although tibial rotational measurements were changed among the different resection levels [23, 24]. Determination of the tibial AP axis based on the bone resection level seems to be unreliable as the rotational reference. In the present study, the tibial AP axis was determined using the preoperative CT image and then three-dimensionally merged with the postoperative CT image using the simulation software. This method allows the accurate measurement of the difference from the preoperative planning in tibial rotational alignment and may reduce the uncertainty in measurement of tibial rotation, although the measurement has complexity and diversity.

The definition of outliers is an important issue when evaluating the accuracy of component positioning in TKA. An outlier of coronal alignment was defined as a mechanical axis with more than 3° varus or valgus in most studies [7, 25, 26], and it seems to be generally accepted. In contrast, there is no agreed definition of the cutoff value for outliers in rotational alignment, especially tibial rotation. This may be due to wide variation in anatomical landmarks and measurement methodology [6]. Although several studies reported various cutoff values for tibial rotation affecting clinical outcomes [5, 27–29], a systematic review identified internal rotation > 10° in the tibial component as a negative prognostic factor [6]. On the basis of these previous reports, internal rotation > 10° was defined as a clinically relevant cutoff value for outliers for tibial rotation in the present study.

PSI has been becoming increasingly popular and is expected to improve surgical performance, including accuracy of component positioning and reduction of outliers. However, the superiority of PSI compared to the conventional technique has been controversial in several systematic reviews [8, 30, 31]. The majority of the reports comparing PSI and conventional technique showed no evidence of superiority in reducing outliers of component alignment, although there is a lack of information on the assessment of tibial rotational alignment [30, 31]. The present study demonstrated that no significant differences were found in coronal alignment, but there was a significant reduction of clinically relevant outliers (internal rotation > 10°) in tibial rotational alignment. The data support previous reports with regard to coronal alignment, but indicate the effectiveness of PSI in rotational positioning of the tibial component.

There are several types of PSI designs, and the procedures of tibial rotational positioning vary. Some PSIs do not directly guide the tibial component positioning, and the rotational positioning is determined in reference to extramedullary rod, AP marker pin, or rotational marker on the PSI. The other types of PSI directly determine the tibial rotational positioning through guide holes located on the tibial plateau, and this type of PSI was used in the present study. The latter types may reproduce the planned tibial rotational positioning better than the former ones, although there are no studies comparing between them.

This study has several limitations. First, this was not a randomized, controlled study but a retrospective, case–control study with an evidence level of III. Second, the patient population was small. Third, the data included only radiographic and 3D-CT measurements, and the relationship with clinical outcomes was not evaluated.

## Conclusion

This study demonstrated that the difference from the preoperative planning in tibial rotational positioning was accurately evaluated using novel three-dimensional measurement method, and PSI could reduce outliers from the planned positioning in rotational alignment of the tibial component (internal rotation > 10°). PSI is a useful technique for improving the reproducibility of the planned tibial rotational positioning in TKA.

## Data Availability

The data and materials used and/or analyzed during the current study are not publicly available but available from the corresponding author on reasonable request.

## Abbreviations

TKA: Total knee arthroplasty
PSI: Patient-specific instrumentation
CT: Computed tomography
AP: Anteroposterior
ICCs: Intraclass correlation coefficients
